# MAVM-UNet: multiscale aggregated vision MambaU-Net for field rice pest detection

**DOI:** 10.3389/fpls.2025.1635310

**Published:** 2025-08-13

**Authors:** Congqi Zhang, Ting Zhang, Guanyu Shang

**Affiliations:** ^1^ School of Software Engineering, Chengdu University of Technology, Chengdu, China; ^2^ School of Computer Science, Xijing University, Xian, China; ^3^ Henan Agricultural Information Data Intelligent Engineering Research Center, Sias University, Zhengzhou, China

**Keywords:** field rice pest detection, Visual State Space (VSS), Channel-Aware VSS (CAVSS), vision MambaU-Net, multiscale aggregated vision MambaU-Net (MAVM-UNet)

## Abstract

Pests in rice fields not only affect the yield and quality of rice but also cause serious ecological and environmental problems due to the heavy reliance on pesticides. Since various pests have irregular and changeable shapes, small sizes, and complex backgrounds, field rice pest detection is an essential prerequisite and challenge for the precise control of pests in the field. A multiscale aggregated vision MambaU-Net (MAVM-UNet) model for rice pest detection is constructed. The model consists of four main modules, Visual State Space (VSS), multiscale VSS (MSVSS), Channel-Aware VSS (CAVSS), and multiscale attention aggregation (MSAA), where VSS is used as the basic module for capturing context information, MSVSS is used to capture and aggregate fine-grained multiscale feature of field rice pest images, CAVSS is added into Skip connection to select the critical channel representations of the encoder and decoder, and MSAA is added in the bottleneck layer to integrate the pest features of different layers of the encoder. Combining MSAA and CAVSS can capture the low-level details and high-level semantics and dynamically adjust the contributions of features at different scales; for example, the slender legs and antennae of pests rely on fine-grained features, while the large body of pests relies on coarse-grained features. A large number of experimental results on the rice pest image subset of the IP102 dataset show that MAVM-UNet is superior to the state-of-the-art models, with *PA* and *MIoU* of 82.07% and 81.48%, respectively. The proposed model provides important guidance for the monitoring and control of pests in rice fields. The codes are available at https://github.com/ZengsihaoNB666/mavmunet.git.

## 1 Introduction

Field crop disease and pest detection (FCDPD) is essential for ensuring the yield and quality of crops. The traditional FCDPD method based on agricultural experts is inefficient and has limited accuracy in large-scale real-time FCDPD (He et al., 2023; Zhang et al., 2023). With the development of Android mobile, Internet of Things (IoT), and unmanned aerial vehicle (UAV) technologies in smart agriculture, many methods for detecting crop diseases and pests have been proposed by easily collecting images of field crops through IoT and UAVs (Saleem et al., 2024; Zhang et al., 2024). In recent years, deep learning models, such as U-Net and its variants, have been widely applied in FCDPD, particularly field crop pest detection (FCPD) (Guo et al., 2024; Saleem et al., 2025). However, due to limited receptive fields, they are unable to extract the long-range dependencies that are crucial for understanding the global context of the field pest image structures (Naeem et al., 2025). Transformer and Vision Transformer (ViT) models have emerged as a promising alternative (Xu et al., 2022; Bai et al., 2023). They are good at capturing long-range dependencies, but not at extracting local features, and their quadratic computational complexity limits their application, especially for high-resolution images of field pests collected by Android mobile, Internet of Things, and unmanned aerial vehicles (Gole et al., 2024; Bedi et al., 2025).

To address the above challenges, Mamba and its improved models have been presented and achieved remarkable development with a linear complexity of *O*(n), including Vision Mamba (VMamba), Mamba-UNet, and MSVM-UNet (Chen et al., 2024; Wang et al., 2024). They excel in capturing long-range dependencies and spatial local features and are particularly suitable for complex FCPD tasks. Aiming at the problems existing in FCPD, such as irregular and variable shapes, blurred pest boundaries, low contrast between pests and the background, and large imaging noise, a multiscale aggregated vision Mamba-UNet (MAMVM-UNet) model for FCPD is constructed. The main contributions of this paper are described as follows:

1. A multiscale Visual State Space (MSVSS) module is proposed to capture and aggregate the multiscale fine-grained features of field crop pest images.
2. A Channel-Aware VSS (CAVSS) Block is added into the Skip connection to incorporate channel-spatial attention features into VSS to select the critical representations of the encoder and decoder.
3. A multiscale attention aggregation (MSAA) module is added to the bottleneck layer to integrate the features of different layers of the encoder.

The rest of this paper is arranged hierarchically, as follows. Section 2 overviews the related work. MAVM-UNet and its components are introduced in detail in Section 3. Section 4 presents the experiments, results, and analyses. The paper is summarized along with prospects for the next work in Section 5.

## 2 Related work

With the advancement of computer vision, IoT, and UAV technologies, many FCPD methods have been continuously presented, which are roughly divided into convolutional neural networks (CNNs), Transformers, Mamba, and their improved models.

### 2.1 CNN-based methods

By stacking deep convolutional layers, CNN can automatically extract useful advanced features from pest images, achieving high performance of FCDPD (Bedi and Gole, 2021a; Bedi and Gole, 2021b). (Wei et al., 2022). proposed a multiscale feature fusion (MFFNet) model for FCPD and integrated multiscale feature extraction and mapping modules to achieve end-to-end precise classification of crop insects. (Zhang et al., 2024). combined the advantages of the attention mechanism and multiscale feature fusion to improve the accuracy of FCPD. They introduced the relationship-aware Global attention module to adaptively adjust the feature weights at each position, pay more attention to the areas related to pests, and reduce background interference. (Wang et al., 2024). constructed a dilated multiscale attention U-Net model for FCPD. In the model, the dilated Inception module replaces the convolution operation in U-Net to extract the multiscale features of pest images, and the attention module focuses on the edges of pest images.

The above models with limited convolutional receptive fields rely on a large dataset for training the model, but it is ineffective for few-shot FCPD and cannot extract the long-range dependencies that are crucial for multiscale FCPD.

### 2.2 Transformer-based methods

In the field of FCPD, Transformer and ViT can analyze the pest behavior changes, providing strong support for the early monitoring (Xie et al., 2024). (Zhang et al., 2023). proposed a multimodal Transformer model for FCPD and obtained more competitive results compared to other excellent models. (Zeng et al., 2024). proposed a lightweight hybrid FCPD network HCFormer, which integrates both the local and global features of the input images, resulting in a more accurate feature representation of crop pests. (Fu et al., 2024). introduced an improved ViT for FCPD. The results indicate that the self-attention mechanism of ViT can optimize the performance of FCPD. (Liu et al., 2025). proposed a Transformer-based end-to-end FCPD method, which can compensate for the feature information loss caused by the downsampling process and achieve remarkable results.

The above analysis demonstrates the powerful performance of Transformers and ViTs in various computer vision tasks, but their quadratic complexity limits their application in high-resolution and real-time FCPD tasks.

### 2.3 Mamba-based method

Mamba and its variants combine state space model (SSM) and Visual State Space (VSS) blocks with advanced deep learning to learn local–global features and remote dependencies, thereby enhancing the performance of image detection and segmentation (Chen et al., 2024; Jiang et al., 2024; Liao et al., 2024; Wang et al., 2024). VSS is regarded as visual SSM. VSS compensates for the inherent deficiencies of SSM in two-dimensional data by introducing spatial serialization strategies, locality modules, and multiscale designs. The essential difference between SSM and VSS lies in the trade-off between universality and domain adaptability. As a visual mamba, VMamba inherits the advantages of CNN and ViTs, improves computational efficiency, and achieves linear complexity without sacrificing the global acceptance field. Mamba-UNet is a hybrid deep learning model that combines U-Net and Mamba. It can effectively capture the global context, significantly improve the accuracy of image detection and segmentation, and maintain relatively low computational overhead. Visual Mamba UNet (VM-UNet) is a hybrid deep learning model that combines U-Net and Mamba to capture global–local features for effective image detection and segmentation. To overcome the limitations of CNN and ViTs (Ruan et al., 2024, Wang et al., 2024). introduced the insect classification model InsectMamba. It integrates SSM, CNN, multi-head self-attention mechanism, and multi-layer perceptron in the hybrid SSM block, achieving an accurate classification of pests.

From the above analysis, it is known that CNNs and ViTs have been widely applied in FCPD. However, CNN is not good at capturing remote dependencies, while the computational complexity of ViT is quadratic. Based on VM-UNet and MSVM-UNet, a multiscale aggregated VM-UNet (MAMVM-UNet) is constructed. The multiscale global context features are captured using VMamba, U-Net, and VM-UNet, and the fine-grained FCPD is achieved by multiscale attention mechanisms.

## 3 The proposed model

**Figure 1:**
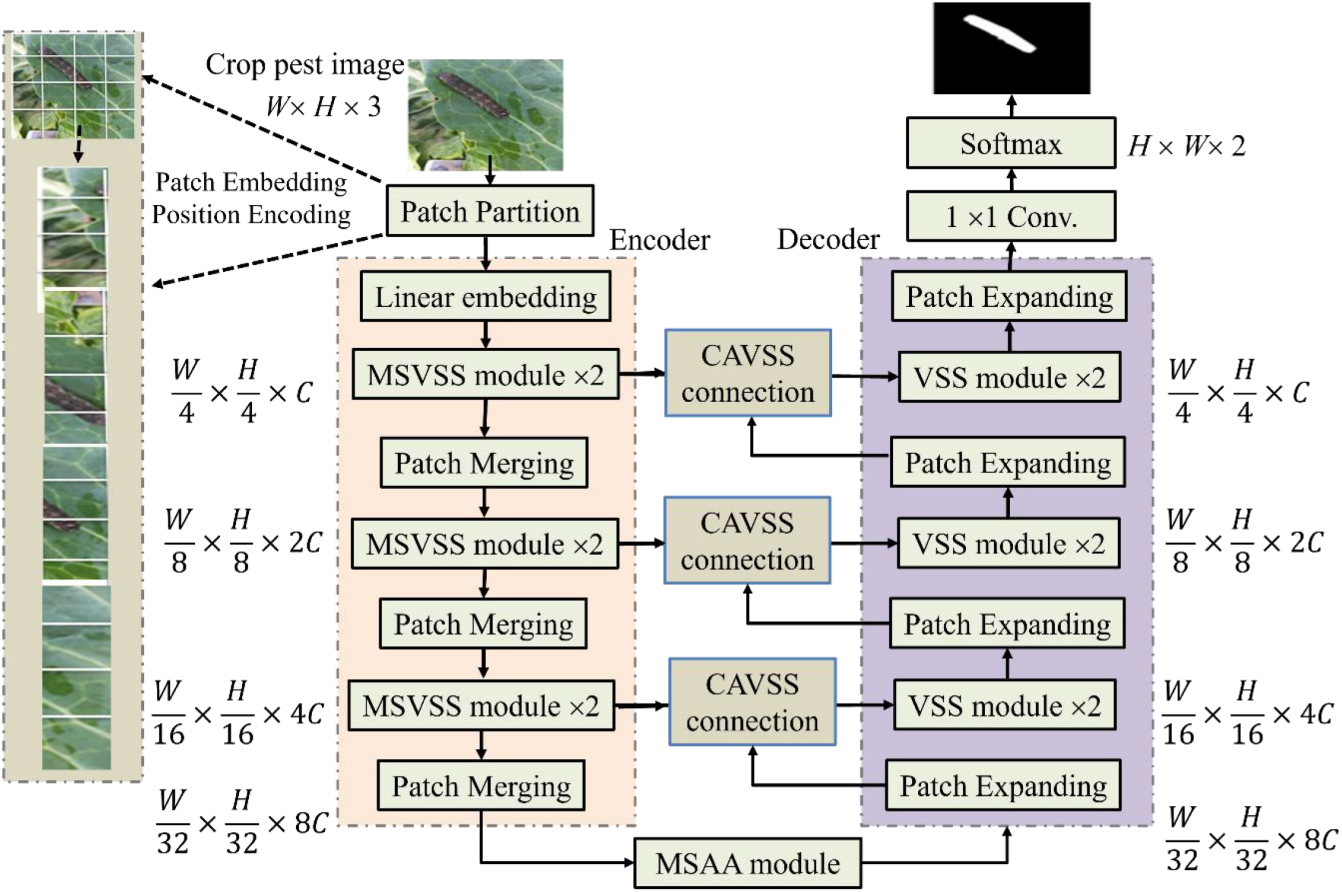
The architecture of MAVM-UNet.

**Figure 2:**
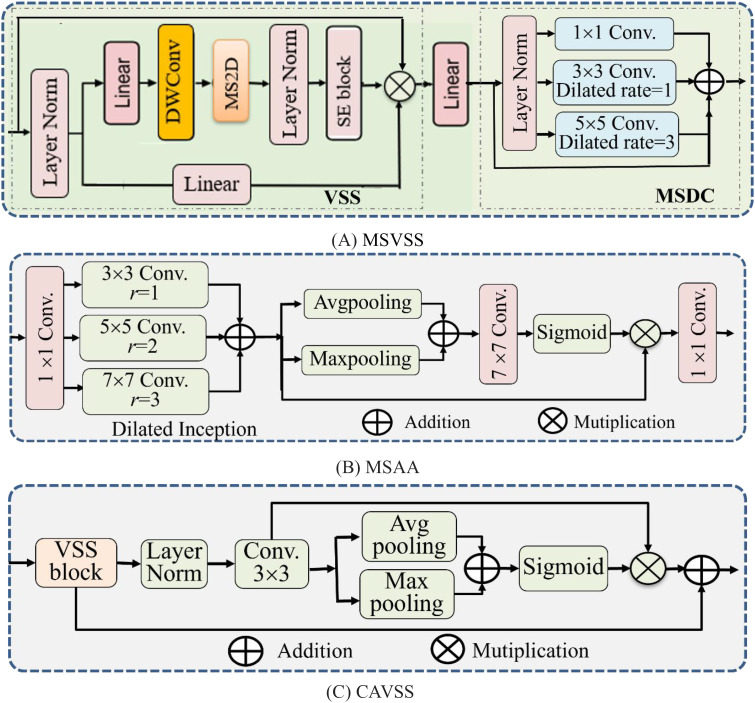
The main components of MAVM-UNet. **(A)** MSVSS. **(B)** MSAA. **(C)** CAVSS. MAVM-UNet, multiscale aggregated vision MambaU-Net; MSVSS, multiscale Visual State Space; MSAA, multiscale attention aggregation; CAVSS, Channel-Aware Visual State Space.

The proposed model MAVM-UNet for FCPD is an improved VM-UNet. Its architecture is shown in **Figure 1**, consisting of an encoder and a decoder, including three main modules: MSVSS, CAVSS, and MSAA. Their structures are illustrated in **Figures 2A–C**, respectively.

### 3.1 Overall architecture of MAVM-UNet

MAVM-UNet is an improved VM-UNet. Similar to ViT and Mamba-UNet, the input $Img\in R^{W\times H\times 3}$ is first split into 16 non-overlapping small patches of 4 × 4 in size in the linear embedding layer and transformed them into a 1D sequence with dimensions of W/4×H/4×16 . Each patch is treated as a token and input into the encoder to learn deep feature representations. In the encoder, after MSVSS, the features are merged by the Patch Merging layer, which is regarded as max-pooling used for downsampling the feature maps. The output resolutions of each layer of the encoder are W/4×H/4×C , W/8×H/8×2C , W/16×H/16×4C , and W/32×H/32×8C , where *C* is the mapping channel dimension default set to 96. Like the encoder, the decoder adopts two consecutive VSS blocks for feature reconstruction, and the Patch Expanding module is used for feature upsampling. It can enhance depth features and improve resolution (by doubling the scale), while halving the feature size, doubling the feature size in the initial layer, and reorganizing and reducing them to enhance resolution. The output resolutions of each layer of the encoder are W/32×H/32×8C , W/16×H/16×4C , W/8×H/8×2C , and W/4×H/4×C . The linear embedding layer adjusts the feature dimension to the size of *C*. The last 1 × 1 convolution is used as a fully connected layer, and Softmax as a classifier is adopted for FCPD.

### 3.2 MSVSS

As shown in **Figure 2A**, MSVSS consists of VSS (**Figure 2A** left) and multiscale dilated convolution (MSDC) (**Figure 2A** right). In VSS, the Selective Scan 2D module (SS2D) is an extension of Mamba (State Space Model) on 2D visual data. It replaces traditional convolution or self-attention through a content-aware sequence scanning mechanism to achieve efficient long-range dependency modeling. It consists of three key components—scan expanding, S6, and scan merging—where scan expanding decomposes the input image into 16 independent sequences along four directions of up, down, left, and right, which can ensure the spatial coverage of information and capture the multi-direction feature; S6 utilizes a selective mechanism to accurately identify and extract useful information while filtering out the irrelevant parts. DWConv is a depthwise-separable convolution module, consisting of Depthwise Convolution and Pointwise Convolution operations. It is often used to reduce the number of weight parameters and computational load by separating the convolution and point-by-point convolution. After VSS, Linear projecting, and Layer norm operations, the output is input into MSDC to perform multiscale dilated convolution with three kernels of sizes 1 × 1, 3 × 3, and 5 × 5. The process of MSVSS is formalized in Equation 1:


(1) $$\begin{array}{l} F_{i+1}^{MSVSS}=MSDC(LP(F_{i}^{VSS}))+LP(F_{i}^{VSS})\\ F_{i}^{VSS}=VSS(LN(F_{i}^{d}))+F_{i}^{d} \end{array}$$

where $F_{i}^{d}$ and $F_{i+1}^{MSVSS}$ are the input feature maps and output feature maps of the *i*th layer of encoder, respectively; $F_{i}^{VSS}$ is the output of the VSS module; *LP*(·) is Linear projecting; *LN*(·) is Layer normalization; and *MSDC*(·) and *VSS*(·) are MSDC and VSS operations, respectively.

In VSS, Squeeze and Excitation (SE) is a lightweight attention module, which can significantly improve model performance with low computational cost by enhancing useful features through adaptive channel weighting and suppressing redundant features.

Three Linear layers are used to realize feature compression, dynamic parameterization, and modal transformation in VSS, which is the key to balancing computational efficiency and modeling ability.

### 3.3 MSAA

MSAA is added to the bottleneck layer of the model, as shown in **Figure 2B**, consisting of a set of dilated convolutions, parallel max-pooling and Avg-pooling, residual connection, and 7 × 7 convolution, followed by Sigmoid activation and 1 × 1 convolution. The dilated convolutions with dilated rates of 1, 3, and 5 are used to capture multiscale features. The process of MSAA is formalized in Equation 2:


(2) $$\begin{array}{l} F^{MSAA}=Conv_{1\times 1}(Sigmoid(Conv_{7\times 7}(Avg(F_{4}^{Dil}),Max(F_{4}^{Dil}))⊗F_{4}^{Dil})\\ F^{Dil}=Conv_{dilated}(Conv_{1\times 1}(F_{4}^{d})) \end{array}$$

where $F_{4}^{d}$ and $F^{MSAA}$ are the input feature maps and output feature maps of MSAA, respectively; $F^{Dil}$ is the output of the Layer norm dilated convolution with dilated rates of 1, 3, and 5; ⊗ is the Hadamard product; and *Avg*(·) and *Max*(·) are parallel max-pooling and Avg-pooling operations, respectively.

### 3.4 CAVSS

CAVSS, as the attentional Skip connection of Mamba-UNet, is used to fuse the multiscale features and upsampled features of the encoder and the decoder together, reducing the loss of spatial information. Its structure is shown in **Figure 2C**, consisting of a VSS block, a Layer norm, a Conv., and two parallel pooling and two residual connections. Its process is formalized in Equation 3:


(3) $$\begin{array}{l} F^{CAVSS}=Sigmoid(Avg(F_{LN}^{VSS}),Max(F_{LN}^{VSS}))⊗F_{LN}^{VSS}+F^{VSS}\\ F_{LN}^{VSS}=Conv(LN(F^{VSS}))\\ F^{VSS}=VSS(F_{i}^{c}) \end{array}$$

where $F_{i}^{c}$ and $F^{CAVSS}$ are the input–output feature maps of CAVSS and *VSS*(·) is the VSS operation.

### 3.5 Patch merging and expanding

Patch Merging in the encoder is regarded as downsampling, and the feature resolution is downsampled by 2×. It retains the global context through Mamba blocks to avoid the information loss of max-pooling in U-Net. Since the concatenating operation results in the feature dimension increasing by 4×, a Linear layer is used to reduce the feature dimension to 2× the original dimension.

Symmetrical to the Patch Merging of the encoder, Patch Expanding in the decoder is used to gradually reconstruct the details of the image and gradually restore the spatial details to achieve precise segmentation. It uses a linear layer on the input features to increase the feature dimension to twice the original dimension, uses a rearrangement operation to expand the resolution of the input features to twice the input resolution, and reduces the feature dimension to 1/4 of the input dimension.

### 3.6 Loss function

The pest image pixels are divided into pest (marked as 1) and background (marked as 0). To address the class imbalance and small pest detection issues, the hybrid loss functions combining Cross-entropy (Ce) $L_{Ce}$ and Dice Loss $L_{Dice}$ is adopted to train MAVM-UNet, calculated as follows:


(4) $$\begin{array}{l} Loss=\lambda L_{Ce}+(1-\lambda )L_{Dice}\\ L_{Dice}=1-2\sum_{c=1}^{C}{\hat{y}}_{c}y_{i}/(\sum_{c=1}^{C}{\hat{y}}_{c}+\sum_{c=1}^{C}y_{i}+\epsilon )\\ L_{Ce}=-\sum_{c=1}^{C}y_{c}\log({\hat{y}}_{c}) \end{array}$$

where $y_{c}$ and ${\hat{y}}_{c}$ are ground truth and predicted probabilities for class *c*, respectively; λ is an adjustment parameter; and ϵ is a very small non-zero number, indicating that proof $L_{Dice}$ is meaningful.

## 4 Experiments and analysis

MAVM-UNet is verified on the rice pest image subset of the public IP102 dataset and is compared with six state-of-the-art models, i.e., combining fuzzy C-Means and gray-level co-occurrence matrix (FCMGLCM) (Chodey and Shariff, 2023), U-Net, DIA-UNet (Zhang et al., 2025), TSRST (Bai et al., 2023), HCFormer (Zeng et al., 2024), Mamba-UNet (Wang et al., 2024), VM-UNet (Ruan et al., 2024), and MSVM-UNet (Chen et al., 2024), where FCMGLCM is a traditional machine learning model, U-Net is the backbone model, DIA-UNet is a multiscale attention U-Net, TSRST and HCFormer are Transformer-based hybrid models, and VM-UNet and MSVM-UNet are two recent improved Mamba-UNet models. They are simply introduced as follows.

➢ FCMGLCM is a hybrid machine learning model that extracts nine statistical texture features for FCPD. FCMGLCM adopts SVM to detect pest pixels without long-term training.
➢ U-Net is the backbone model.
➢ DIA-UNet is a dilated Inception attention U-Net.
➢ TSRST is a hybrid lightweight model combining Transformer and Super-Resolution Sampling Techniques.
➢ HCFormer is a lightweight FCPD model combining CNN and ViT.
➢ Mamba-UNet is the backbone model of VM-UNet, MSVM-UNet, and the proposed model.
➢ VM-UNet is a vision Mamba-UNet using VSS to capture contextual information with low calculation cost.
➢ MSVM-UNet is a multiscale VM-UNet by multiscale VSS blocks to effectively capture and aggregate the multiscale feature representations from the hierarchical features of the VMamba encoder and better handle 2D visual data.

### 4.1 Dataset

**Figure 3:**
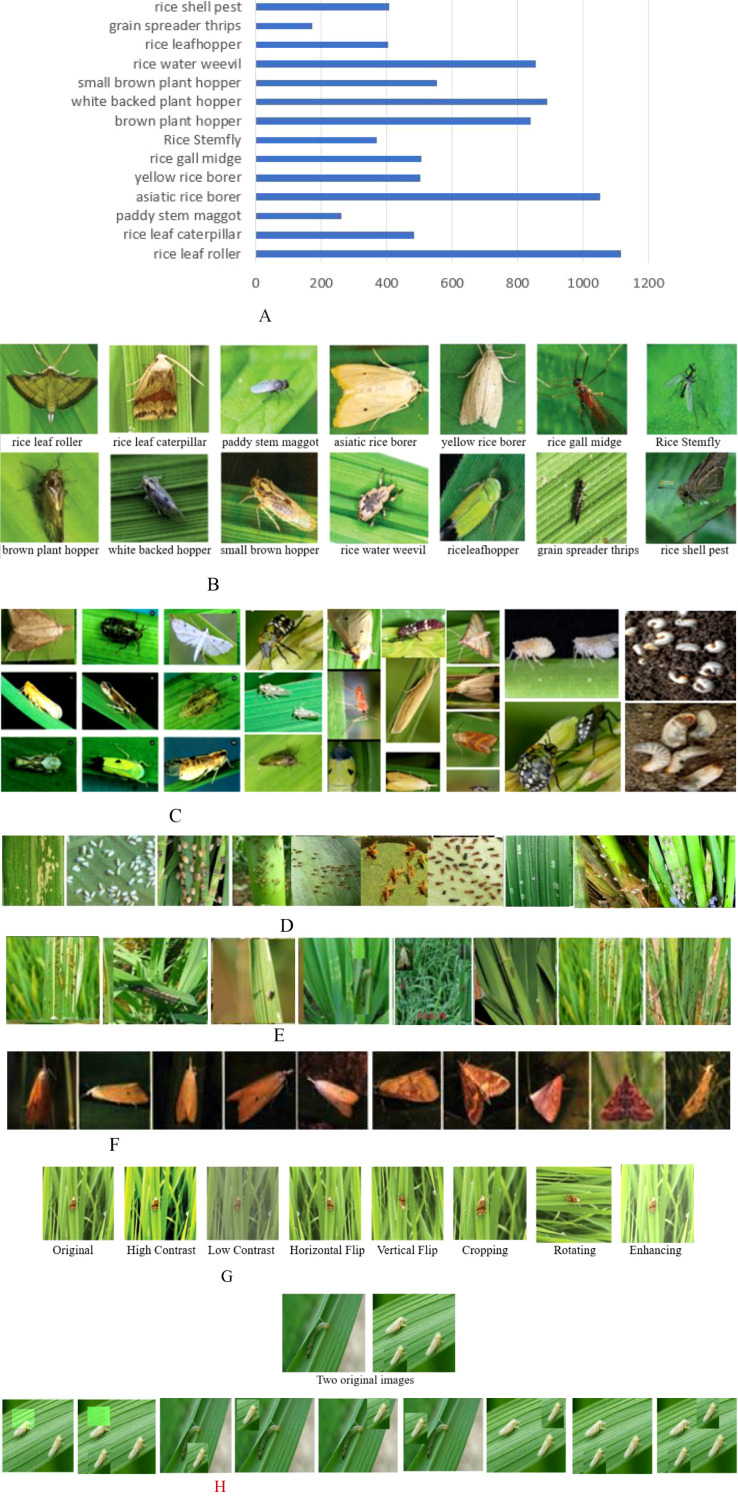
The image number, rice pest image, and augmented image examples. **(A)** The number of pest images. **(B)** Fourteen pest images, one image per category. **(C)** Various rice pests with different shapes and sizes. **(D)** Many fine rice pests. **(E)** Not obvious rice pests. **(F)** Ten images of an asiatic rice borer with different shapes and sizes. **(G)** Seven augmented images of one on the far left. **(H)** Nine augmented images by Mixup and CutMix. MAVM-UNet, multiscale aggregated vision MambaU-Net.

IP102 is a large public image dataset (https://github.com/xpwu95/IP102) (Wu et al., 2019). It has 75,222 images distributed across various crops and environments, covering 102 common pests of eight crops, including rice, alfalfa, wheat, corn, grapes, sugar beets, citrus, and mangoes. Their indices and names can be obtained from https://github.com/xpwu95/IP102/blob/master/classes.txt. IP102 has 8,417 images of 14 types of rice pests. The number of pest images and some examples are shown in **Figure 3**. **Figure 3A** illustrates the names of rice pests with the image counts of each category, and **Figure 3B** shows 14 images, one image per category. **Figures 3C–E** show various pest images with different shapes, colors, and backgrounds in the field. **Figure 3F** shows some images of a pest at its different growth stages. **Figures 3B–F** exhibit distinct appearance characteristics at different growth stages, while different species of insects share similar characteristics.

**Figure 3A** shows that the subsets of images of rice pests are highly unbalanced. For example, there are 1,115 images of rice leaf rollers and 173 images of rice-rice thrips. Imbalance may lead to bias and overfitting in FCPD. To solve this problem of insufficient training samples, some image augmentation algorithms are adopted to generate more images, such as randomly cropping, left and right flipping, up and down flipping, enhancing, random rotating, and random shifting. **Figure 3G** shows seven augmented images of an original image. Mixup and CutMix are two data augmentation techniques, and their main difference lies in the way they are mixed. MixUp is for smoothing decision boundaries (e.g., classification), and CutMix is for localization tasks (e.g., object detection). Nine augmented images are shown in **Figure 3H**. In the following experiments, we only randomly select some augmented images so that there are at least 500 images for each category of the 14 types of rice pests. Then, an augmented dataset containing 9,314 images of rice pests is constructed, including 8,515 original and 799 augmented images.

### 4.2 Experimental set

All models except GLCM are performed on Intel Core i9-10900K CPU, CUDA 11.8, and Nvidia GeForce RTX 3090 GPU using Python 3.8.8 and PyTorch 1.10 framework. The hyperparameters of each model are initialized by random variables of a normal distribution and are optimized by Stochastic Gradient Descent (SGD). In Equation 4, λ is set as 0.4, and ϵ is set as 0.001. The model training parameters are set as shown in **Table 1**.

**Table 1:** The experimental set of the model.

Name	Set
The number of training Iterations	3,000
Batch size	24
Initialized learning rate	0.001
momentum	0.9
Weight decay	0.0001

The model performance is evaluated on the validation dataset every 200 iterations, and the model weights are saved only upon achieving a new best performance on the validation subset. Pixel accuracy (*PA*) and Mean *IoU* (*MIoU*) are commonly adopted to evaluate the performance of the image object detection and semantic segmentation models by the similarity between the predicted result and the ground truth, calculated in Equation 5:


(5) $$\begin{array}{l} PA=\sum_{i=1}^{n}N_{ii}/(\sum_{i=1}^{n}\;\sum_{j=1}^{n}N_{ij})\\ MIoU=\frac{1}{n}\sum_{i=1}^{n}N_{ii}/(\sum_{j=1}^{n}N_{ij}+\sum_{j=1}^{n}N_{ji}-N_{ii}) \end{array}$$

where *n* is the number of categories, $N_{ii}$ is the pixel number of true positives, $N_{ij}$ is the total pixel number of the *i*th true category predicted as the *j*th category, and $N_{ij}$ and $N_{ji}$ are the pixel number of false positives and false negatives, respectively.

All 9,314 images of rice pests in the augmented dataset are divided into three subsets in a ratio of 6:2:2, of which approximately 60% is used for training, 20% for verification, and the remaining 20% for testing to obtain the detection result. After repeating this 6:2:2 experiment 50 times, the average results of *PA* and *MIoU* are calculated as the FCPD results.

To be fair in the experiments, all models except FCMGLCM are performed using the above dataset and data preprocessing algorithms under the same optimizer and the same loss function. FCMGLCM is conducted by feature extraction and SVM. MAVM-UNet and the baseline models are trained under consistent settings, with hyperparameters tuned via an evaluation metric standardized across all comparisons. Pretrained weights are used where applicable (e.g., for backbone architectures like U-Net and VMamba) as noted in Res (Bai et al., 2023; Chen et al., 2024).

### 4.3 Visualization

**Figure 4:**
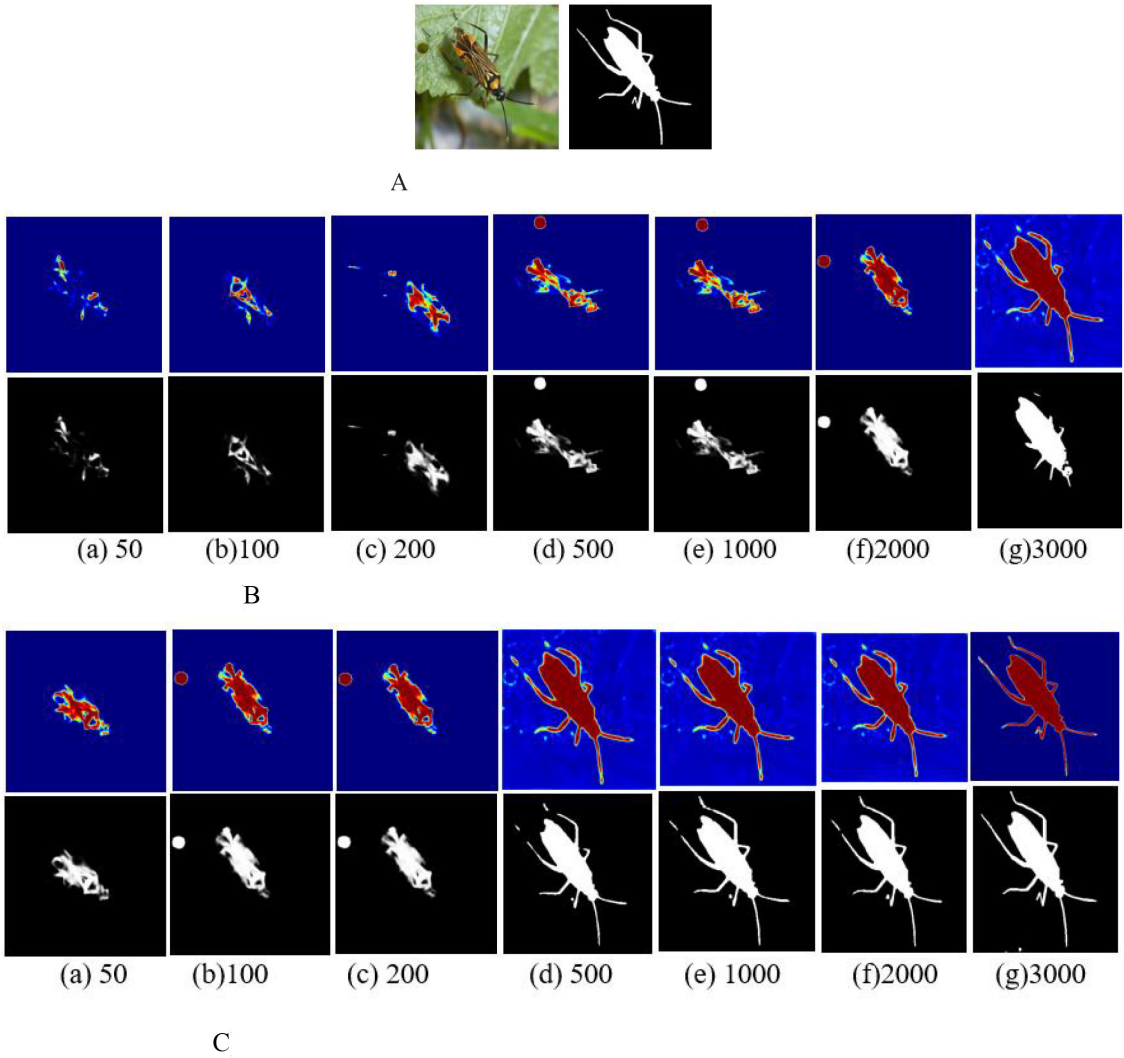
The hot maps and detected pests versus the number of iterations. **(A)** Original and labeled image. **(B)** The hot maps and corresponding detected pests by U-Net. **(C)** The hot maps and corresponding detected pests by MAVM-UNet. MAVM-UNet, multiscale aggregated vision MambaU-Net.

To verify the model performance of MAVM-UNet, **Figure 4** illustrates the hot maps, compared with U-Net. **Figure 4** shows that MAVM-UNet can capture the salient features of the pest, and it can detect the pests with complete shapes and edges at the 1,000th iteration, while the detected pests are complete by U-Net at the 2,000th iteration. The detected results indicate that MAVM-UNet is more stable and faster to converge than U-Net.

**Figure 5:**
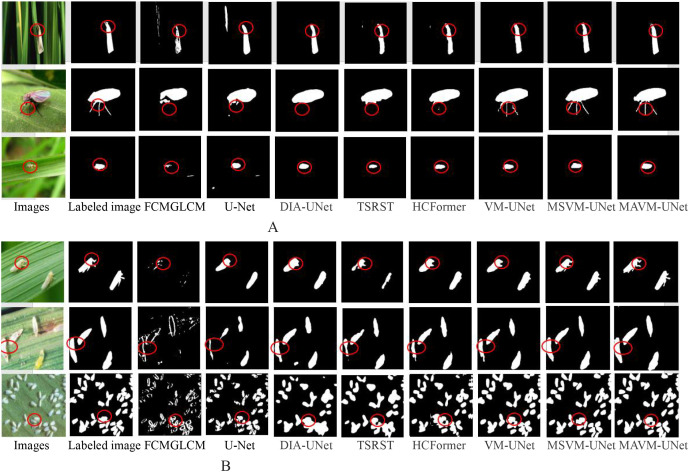
The detected pests from three simple images and three complex images. **(A)** One pest in an image. **(B)** Various multiscale pests in an image.

To visually compare the pests detected by the proposed model MAVM-UNet and the seven comparison models, **Figure 5** illustrates six randomly selected original images (three simple images, each with only one pest, and three complex images, each with several multi-shape pests), the corresponding labeled images, and the pest images detected by the seven comparison models and MAMM-UNet.

**Figure 5** clearly shows that FCMGLCM cannot effectively detect field pests because it is difficult to extract the optimal classification features from the images of rice pests in the field, resulting in poor detection performance of field pests. It is also found that MAVM-UNet is superior to the other seven models, and DIA-UNet is slightly superior to U-Net because the max-pooling of U-Net may lead to detailed information loss.

**Figure 4A** shows that, except for FCMGLCM and U-Net, all models can satisfactorily obtain the complete contour of each pest from the simple images, and MSVM-UNet and MSVM-UNet can obtain complete and thin legs of the pest. **Figure 4B** shows that U-Net and DIA-UNet are more likely to over-detect pests (in the fourth and fifth columns), TSRST and HCFormer under-detect the pests (in the sixth and seventh columns), MAVM-UNet, VM-UNet, and MSVM-UNet can detect fine-sized pests in a dense distribution. The results indicate that the models based on VMamba architecture have a stronger ability to encode the global context and distinguish semantics. **Figure 4B** also shows in the third column that the dense fine pests cannot be detected by FCMGLCM. The reason is that FCMGLCM cannot correctly extract the features from the various poses and shapes of the dense fine pests, resulting in poor detection performance.

### 4.4 Quantitative results

The proposed model is further verified through a series of 6:2:2 experiments and is quantitatively compared with the seven comparison models. **Table 2** presents the pixel accuracy (*PA*), *MIoU*, model training time, and GFLOPs (Giga Floating-point Operations Per Second) of eight models.

**Table 2:** The detection results of eight models.

Results Models	*PA* (%)	*MIoU* (%)	Training time (h)	GFLOPs
FCMGLCM	58.80	56.28	0.50	8
U-Net	69.22	68.29	6.39	75
DIA-UNet	72.20	71.07	6.88	98
TSRST	74.08	73.14	8.48	90
HCFormer	75.28	75.12	8.42	95
VM-UNet	75.85	75.70	3.27	55
MSVM-Mamba	80.24	77.32	3.36	70
MAVM-UNet	82.07	81.48	3.30	58

GFLOPs, Giga Floating-point Operations Per Second; FCMGLCM, fuzzy C-Means and gray-level co-occurrence matrix; MAVM-UNet, multiscale aggregated vision MambaU-Net.

**Table 2** shows that the *PA* and *MIoU* of MAVM-UNet are the highest at 82.07% and 81.48%, respectively, but MAVM-UNet’s training time and GFLOPs are slightly longer than those of VM-UNet due to the relatively time-consuming nature of MSAA and CAVSS modules. MSVM-Mamba is better than VM-UNet because it is a multiscale VM-UNet and is effective for multiscale pest detection. The result of FCMGLCM is the lowest, but its training time is the least because it relies on the handcrafted features and only needs to train the SVM classifier. The result of U-Net is the second lowest due to its max-pooling, leading to the inaccurate detection and location of pests by this model. TSRST and HCFormer are better than DIA-UNet, but their training time is the longest. The reason is that they can obtain the global contextual features from the complex field pest images, while the computational complexity of their backbone network Transformer is quadratic.

### 4.5 Ablation experiments

**Figure 6:**
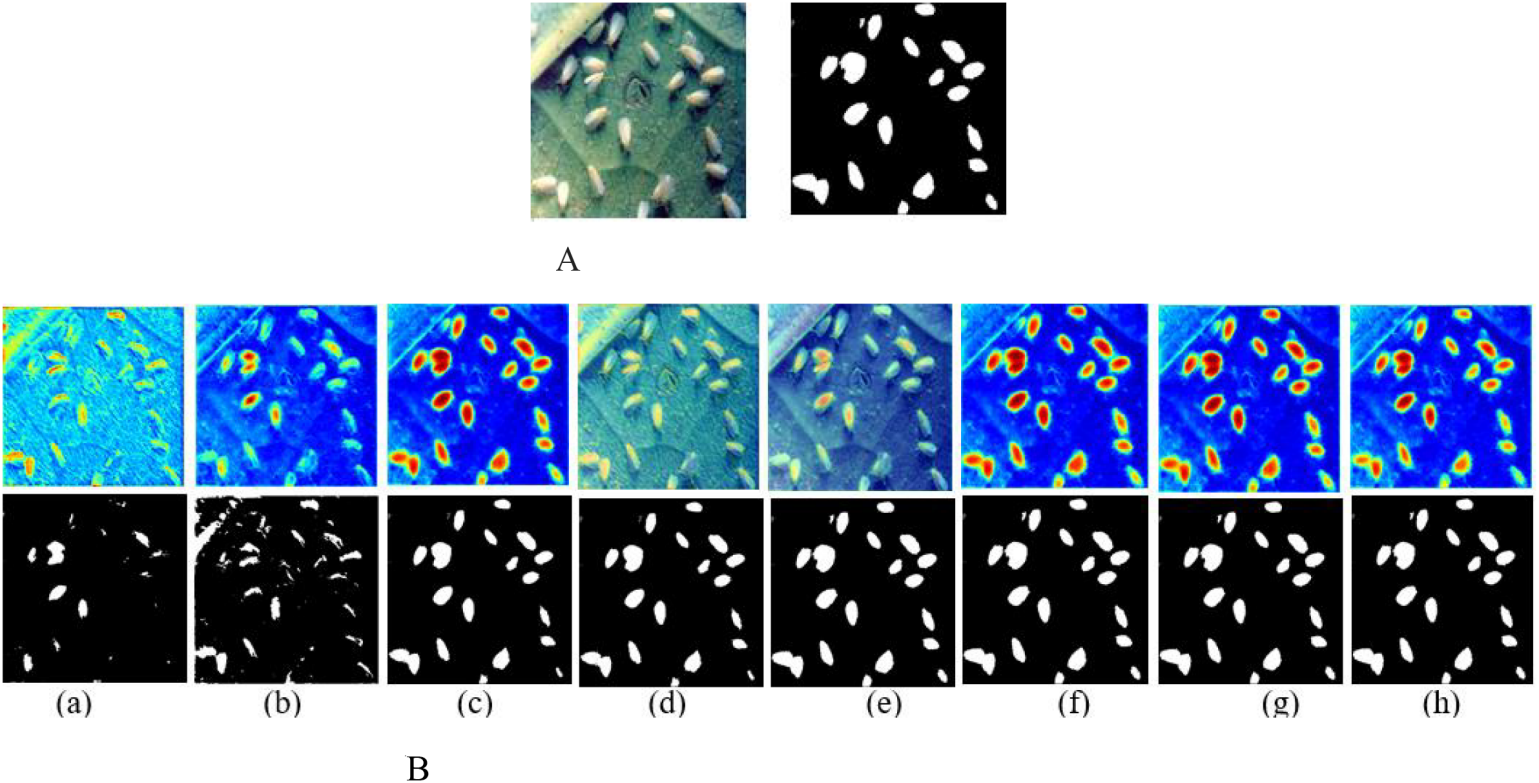
The hot maps and detected pests by the variants of MAVM-UNet, where (a) without MSAA, MSVSS, and CAVSS;, (b) without MSAA and CAVSS;, (c) without MSAA and MSVSS;, (e) CAVSS is replaced by channel-spatialChannel-Spatial attention;, (f) MASS is replaced by VSS;, (g) MASS is replaced by CBAM (Convolutional Block Attention Module (CBAM);, and (h) MSVSS in encoder and VSS in decoder are replaced by lightweight Transformer. **(A)** Original and labeled image. **(B)** The hot maps and corresponding detected pests. MAVM-UNet, multiscale aggregated vision MambaU-Net; MSVSS, multiscale Visual State Space; MSAA, multiscale attention aggregation; CAVSS, Channel-Aware Visual State Space.

MAVM-UNet is an improved model of VM-UNet. The main improvements are three modules: MSVSS, MSAA, and CAVSS. To verify the robustness of MAVM-UNet, a series of 6:2:2 experiments are conducted to investigate the impact of MSAA and CAVSS on the performance of FCPD under the same experimental conditions mentioned above. The detected pest images are shown in **Figure 6**. **Table 3** presents the quantitative detection results of these experiments.

**Table 3:** The experiment results by variants of MAVM-UNet.

Results Variants	*PA* (%)	*MIoU* (%)	Training time (h)
(a) Without MSAA, MSVSS, and CAVSS	68.52	67.38	3.64
(b) Without MSAA and CAVSS	73.68	71.32	3.51
(c) Without MSAA and MSVSS	76.43	74.46	3.29
(d) MSVSS in encoder is replaced by VSS	79.19	78.27	3.17
(e) CAVSS is replaced by channel-spatial attention	81.15	80.66	3.25
(f) Dilated conv. module in MSDC of MSVSS is replaced by DWConv	81.36	80.60	3.22
(g) MASS is replaced by CBAM	81.10	80.18	3.27
(h) MSVSS in encoder and VSS in decoder are replaced by lightweight Transformer	81.72	81.26	7.11

MSAA, multiscale attention aggregation; MSVSS, multiscale Visual State Space; CAVSS, Channel-Aware Visual State Space; MAVM-UNet, multiscale aggregated vision MambaU-Net; CBAM, Convolutional Block Attention Module.

**Figure 6** and **Table 3** show that MSVSS, MSAA, and CAVSS are three very important modules that can improve the detection performance of the model. The reason is that MSVSS and MSAA can obtain multiscale features, which are superior to the direct image division in VM-UNet. Compared with VSS in VM-UNet, MSVSS enhances the performance of extracting deep semantic features of the images of the original SSM blocks. Adding MASS to the bottleneck layer can improve the detection results, but the training time is slightly longer. The lightweight Transformer block can enhance global feature extraction while increasing large computational cost. **Figure 6** and **Table 3** indicate that MAVM-UNet can achieve remarkable results for small-sized and densely distributed pests in rice fields.

To check the effect of data augmentation on pest detection performance, some experiments are implemented on the original dataset and the augmented dataset. The results are given in **Table 4**. **Table 4** shows that the PA and training time are improved on the augmented dataset. The main reason is that the IP102 dataset is class-imbalanced, and data augmentation can avoid overfitting and vanishing gradients. The training time on the original dataset is longer than that on the augmented dataset because the original dataset is insufficient to simulate the characteristics of pests.

**Table 4:** The effect of data augmentation on pest detection performance.

Results Dataset	*PA* (%)	*MIoU* (%)	Training time (h)
Original dataset	81.61	80.27	4.28
Augmented dataset	82.07	81.48	3.30

### 4.6 Analysis

The field pest images are complex with small-sized and various-shaped pests, and many models find it difficult to capture the features of dense fine pests. VM-UNet is an effective backbone network for various segmentation and detection tasks in computer vision and can solve the problem of long-range dependency modeling caused by the inherent locality of U-Net and the computational complexity of Transformer. MAVM-UNet is an improved model of VM-UNet. **Figures 4**-**6** and **Tables 2**-**4** show that when the pests are very small with various shape details and there is low contrast between the pests and the background, MAVM-UNet, VM-UNet, and MSVM-UNet can detect field pests and are generally superior to other models. For smaller pests with thinner antennae and legs, MAVM-UNet can also locate pests and obtain pest details more accurately and precisely, while other models have phenomena such as missed detections and incompleteness to different degrees. The above results verify that the constructed MAVM-UNet outperforms the state-of-the-art models. The training time and GFLOPs of the proposed model are slightly longer than those of VM-UNet because the proposed model is more complex than VM-UNet.

## 5 Conclusions

The detection of pests in rice fields is important for the timely prevention and control of pests in rice. However, since the collected images of field pests are often complex and irregular with a massive background, pest detection is still a challenging task. Mamba-UNet can overcome the limitations by effectively capturing long-range dependencies with linear computational complexity through the utilization of the selective structure state space model. Inspired by VM-UNet and MSVM-UNet, a multiscale aggregated vision MambaU-Net (MAVM-UNet) is constructed. The model consists of three main modules: MSVSS, MSAA, and CAVSS. By integrating MSVSS, MSAA, and CAVSS, MAVM-UNet can effectively capture the multiscale contextual global–local features and long-range dependencies of various pests in rice fields. Experimental results on the rice pest image subset of the IP102 dataset indicate that the proposed MAVM-UNet is effective for field rice pest detection, with *PA* and *MIoU* of 82.04% and 81.37%, respectively. This model provides technical support for a pest and disease detection system by unmanned aerial vehicle equipment and an IoT platform. Future work should include trimming the model. Removing redundant neurons or layers can reduce the model size while maintaining performance. Designing a downsized version of VMM-UNET (for example, with fewer channels or a shallower architecture) can make it suitable for edge devices.

## Data Availability

The original contributions presented in the study are included in the article/supplementary material. Further inquiries can be directed to the corresponding author.
